# Novel miRNA-mRNA interactions conserved in essential cancer pathways

**DOI:** 10.1038/srep46101

**Published:** 2017-04-07

**Authors:** Eduardo Andrés-León, Ildefonso Cases, Sergio Alonso, Ana M. Rojas

**Affiliations:** 1 Computational Biology and Bioinformatics Group, Institute of Biomedicine of Seville, HUVR/US/CSIC, 41013 Sevilla, Spain; 2 REDgene Bioinformatics, Sevilla, Spain; 3 Program of Predictive and Personalized Medicine of Cancer (PMPPC), Germans Trias I Pujol Research Institute (IGTP), Can Ruti Campus, Badalona 08916, Barcelona, Spain

## Abstract

Cancer is a complex disease in which unrestrained cell proliferation results in tumour development. Extensive research into the molecular mechanisms underlying tumorigenesis has led to the characterization of oncogenes and tumour suppressors that are key elements in cancer growth and progression, as well as that of other important elements like microRNAs. These genes and miRNAs appear to be constitutively deregulated in cancer. To identify signatures of miRNA-mRNA interactions potentially conserved in essential cancer pathways, we have conducted an integrative analysis of transcriptomic data, also taking into account methylation and copy number alterations. We analysed 18,605 raw transcriptome samples from The Cancer Genome Atlas covering 15 of the most common types of human tumours. From this global transcriptome study, we recovered known cancer-associated miRNA-targets and importantly, we identified new potential targets from miRNA families, also analysing the phenotypic outcomes of these genes/mRNAs in terms of survival. Further analyses could lead to novel approaches in cancer therapy.

Unrestrained cell proliferation is the principal hallmark of cancer, provoked by DNA insults and other events. These insults produce mutations in genes that might alter their expression and/or function, deregulating many physiological pathways and provoke chromosomal damage, events that drive oncogenic transformation and tumour progression^1^. A physiological response is triggered by cells to restrain genomic instability by removing or repairing DNA lesions, widely known as the DNA damage response pathway (DDR)^2^. Different levels of damage produce different responses, such that mild DNA damage induces a reparative response, while severe damage activates cell death in a regulated manner. Distinct proteins are involved in these processes. For instance, p53 contributes to the differential expression of pro-survival and pro-apoptosis genes, while ATM/ATR^3^ promotes cell death when there are many double strand breaks (DSB), activating CASP2, E2F1, P73 or CHEK1. In addition, several pathways are also relevant to restrain oncogenesis, by activating or repressing genes that control cell proliferation and programmed cell death. In particular, the pathways controlling apoptosis, necrosis and senescence are of special interest, as are those involved in the DDR, the cell cycle, DNA replication and telomere elongation, all of which constitute an intricate network in which there is extensive cross-talk that influences events like cancer initiation and progression^4^. Indeed, the intense research carried out into these pathways has helped characterize a large number of mechanisms directly implicated in tumour development^5^.

To further our understanding of cancer and to develop new treatments, several international initiatives have set out to generate complete libraries of tumour types. For example, The Cancer Genome Atlas (TCGA) consortium provides information extracted from a wide variety of cancers using different techniques, housing datasets containing DNA methylation samples, whole genome and whole exome sequences, transcriptome data (RNASeq and small RNASeq), etc. Previous studies identified genes related to cancer malignancy by comparing tumour tissue with normal tissue samples^6^. Such studies yielded relevant genes, of which oncogenes and tumour suppressor genes received most attention as they have the potential to convert a normal cell into a cancer cell. Oncogenes are often mutated or expressed at high levels in tumour cells, while tumour suppressor genes may also be mutated or expressed more weakly than normal. *TP53*^7^ or *FOS*^8^ are examples of tumour suppressor genes where mutations, deletions and/or repression are implicated in processes related to cancer development. Similarly, the oncogenes *E2F3*^3^ and *AURKA*^9^ induce cell growth and proliferation when mutated or over-expressed, driving cells through the cell cycle checkpoints.

In the past decade, the role of microRNAs (miRNAs) in cancer and in cell proliferation has gained significance given their critical role in regulating target genes. These are single stranded non-coding RNAs of 19–22 nucleotides commonly involved in mRNA destabilization and degradation^10^,^11^. miRNAs from the same family share a high degree of sequence homology and they are commonly found in clusters, the components of which are expressed simultaneously, in turn resulting in a tendency to regulate genes with similar functions^12^. Thus, the inappropriate expression of miRNAs that regulate key genes like oncogenes or tumour repressor genes can lead to tumour development, and families such as miR-15, miR-1 and let-7 have frequently been related with cancer growth and metastasis.

The specific miRNAs that are capable of transforming normal cells into tumour cells are known as oncomiRs. For instance, the miR-17/92 cluster, a polycistron RNA that encodes for 6 different miRNAs, was the first oncomiR described. Its over-expression is related to *E2F1*^13^ and *AIB1*^14^ down regulation, which in turn produces tumours by inducing cell growth and proliferation. Conversely, there is another subset of miRNAs, the tumour suppressor miRNAs or anti-oncomiRs, among which we can distinguish the miR-143/145 cluster that is transcribed as a bicistronic unit. Relative to normal cells, these miRNAs are expressed more weakly in colon cancer cells and in breast, lung, head and neck, and bladder cancers^15^. The miR-143/145 cluster targets a large number of genes and their deregulation is thought to be one of the earliest events in cancer development^16^.

The relationships between miRNAs and their mRNA targets are difficult to study experimentally. Current methods are complex, expensive and must overcome technical challenges like tissue specificity, weak expression, 3′ UTR selection and miRNA stabilization^17^. Some techniques measure mRNA expression upon the modification of miRNA expression using luciferase assays or other proteomic assays like pulsed stable isotope labelling with amino acids in cell culture (pSILAC). These approaches serve to quantify the protein derived from the mRNA regulated by different levels of miRNA^18^. Given the known level of complexity, computational predictions of putative miRNA-mRNA targets emerge as a complementary approach to facilitate the experimental characterization of relevant miRNAs. The most frequent procedures are based on seed sequence complementarity, evolutionary conservation and thermodynamic stability^19^. Moreover, additional steps can be implemented to reduce the unavoidable number of false positives obtained from software prediction. In this regard, miRNA target prediction can be improved by using inverse correlated profiles from over-expressed miRNAs and down-regulated genes from the same tumour samples, and vice versa^20^.

One relatively unexplored issue is the potential identification of specific miRNA-mRNA interactions within well-established cancer pathways that are amenable for therapeutic targeting. To investigate whether such interactions are associated with cancer, an integrated analysis of several different tumour types within a comprehensive statistical framework could aid the discovery of specific cancer related miRNA-mRNA signatures. In addition, the analyses of these signatures in different tumours, accounting for additional factors like gene methylation (MET) and copy number alterations (CNAs), could provide relevant information regarding the signature’s stability. In the context of survival, such information is likely to be useful to define novel therapeutic strategies^21^.

## Results

### Genome-wide analysis of individual tumours

Thousands of samples were examined for each of the 15 tumour types analysed here (see Supplementary Table 1 for details), performing differential expression analyses to identify deregulated genes and miRNAs. Only those exhibiting reliable FDR values (FDR < 0.05) and significantly altered expression (absolute log2FC > 1) were selected for further analysis. Details regarding the analysis of RNASeq and small RNASeq samples from each individual tumour type are described in the Supplementary Text.

### Integrative data analysis for the set of 15 tumours

**Figure 1:**
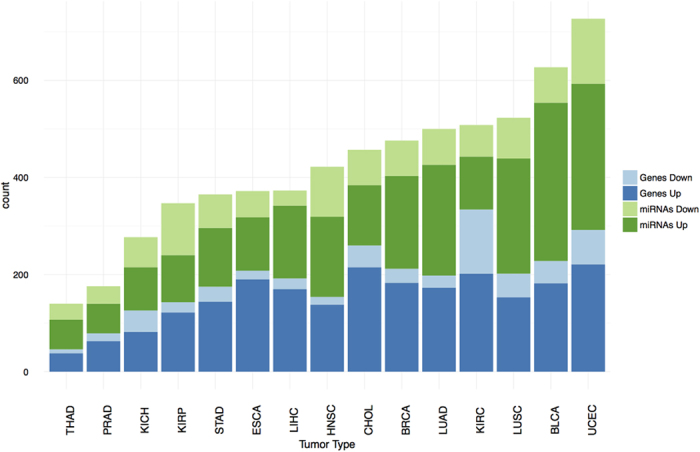
Number of differentially expressed genes and miRNAs (over-expressed and repressed) in each tumour type.

We performed a comprehensive statistical analysis on a set of 15 tumours to identify novel miRNA-mRNA associations that might commonly be deregulated. To this end, we extracted data on differentially expressed genes and miRNAs, comparing samples from healthy subjects and tumour patients from each tumour (see Fig. 1 for the number of significantly deregulated genes and miRNAs).

#### Pathway enrichment

**Figure 2:**
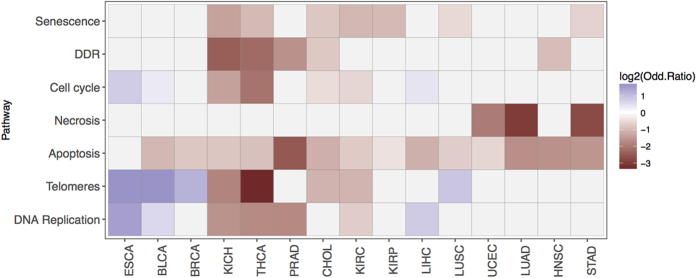
Pathway enrichment analysis of the differentially expressed genes in each tumour type. Statistical significance threshold was set at P < 0.05. Enrichment is indicated by log2 of the odd ratio. In blue, positively enriched pathways; in red, negatively enriched pathways (more altered than expected and less altered, respectively).

In order to estimate the deregulation of a pathway as a whole, we adopted the net effect of all the genes in a pathway rather than the individual contribution of only a few genes. To this end, deregulated genes from each tumour type were extracted and pathway enrichment was studied. Consequently, we inferred that pathways differed mildly across the distinct tumour types (Fig. 2). However, when the pathways were compared in all the cancer types, we observed that significantly altered pathways known to be related to cancer inhibition (apoptosis, necrosis and senescence, or DDR) frequently appeared to be depleted in differentially expressed genes. Adopting this approach means that genes may exhibit the opposite tendency in terms of expression to that of the pathway as a whole. For instance, although *TP53* is up-regulated in CHOL, de-regulation of the remaining genes in the pathway produce a net silencing of the pathway in this particular cancer. On the other hand, *TP53* is down-regulated in several tumour types (BRCA, ESCA, KIRP, LUAD, LUSC, and PRAD) in agreement with our observations. Indeed, while certain genes may frequently be mutated in cancer, in some cases these mutated forms are not functional. One such example is the aforementioned *TP53*, which is often over-expressed in cancer when it carries mutations, although many of these overexpressed mutated variants have lost their original function^22^. As such, non-functional overexpressed forms of *TP53* are not capable of triggering apoptosis. We observed a similar trend for other genes and while we found *MDM2* to be up-regulated in most tumours (Supplementary Table 6), the overall senescence pathway was dampened (Fig. 2). The same applies to *RB1*, which is up-regulated in 2 tumour types (CHOL and HNSC) but it is down-regulated in another 4 tumours (BLCA, LIHC, LUAD, LUSC), consistent with the effect on the whole pathway. Other pathways exhibited distinct trends depending exclusively on the tumour type, such as DNA replication, telomere elongation and cell cycle (more details are available in Supplementary Table 2).

#### Genes differentially expressed across all tumour types

Significantly deregulated genes were retrieved from all types of tumours (for details see Supplementary Text), yet in keeping with other procedures^23^,^24^, only those genes differentially expressed in at least 8 different tumour types were considered for further analysis. This cut-off was established as it reflected the best combination of tumour types, and the best enrichment of cancer-related genes and miRNA-related genes. To this end we compiled known cancer genes from COSMIC^25^ and the network of cancer genes (NCG 5.0 26), which offers a comprehensive list of known cancer genes (more than 600). Using different thresholds for the number of tumour types (from 5 to 10), the figure of 8 tumours appeared to be the cut-off with the highest odds ratio (enrichment in known cancer genes) with a significant right-tailed Fisher false discovery rate (FDR) of 1.54E-02 for COSMIC and 1.87E-03 for NCG (for more details, see Supplementary Tables 3 and 4).

**Figure 3:**
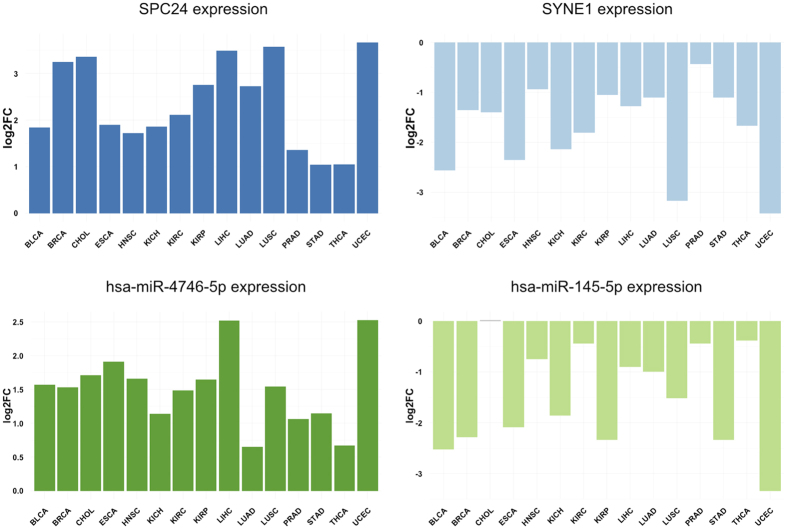
Genes and miRNAs deregulated almost all of the studied tumour types: *SPC24* is differentially overexpressed in all tumour types; *SYNE1* is repressed in all tumour types; miR-4746-5p up-regulated in all the tumour types; and miR-145-5p, down-regulated in all tumour types except Cholangiocarcinoma (CHOL).

From the total of 147 significantly deregulated genes (see Supplementary Table 6), we identified *SCP24* as the only gene over-expressed in all tumour types. We also found *SYNE1* (relevant to the cell cycle) to be the most strongly downregulated gene in the 15 tumours studied (see Fig. 3 for the distribution of both *SPC24* and *SYNE1*). From the remaining differentially expressed genes, 11 belonged to the apoptosis pathway, in which *E2F1* and *CLSPN* were deregulated in 14 and 12 tumour types, respectively. In other pathways, 126 genes appeared to be misregulated in cell cycle pathways and 21 were altered in the DDR, where the oncogenes *CDT1, PLK1* and *DTL* were among the most prominent deregulated genes. In the DNA replication pathway, 26 genes were de-regulated including the previously identified *E2F1, CDT1* and the oncogene *GINS2* as the most significantly affected genes. Of the 29 genes for senescence that were deregulated, *Ubhc10*/*UBE2C* was the most strongly deregulated. Finally, the polymerases *DNA2* and *POLE2* appeared to be deregulated in the telomere pathway in a large number of tumours.

#### miRNAs differentially expressed in tumours

We identified 95 miRNAs that were significantly deregulated in at least 8 of the 15 different tumour types studied (see Supplementary Table 7). As indicated above, this cut-off was selected by performing enrichment at different thresholds using data from the cancer related miRNAs stored in OncomirDB^27^, a database that is a high-confidence reference resource for studying miRNAs related to cancer. Among the up-regulated miRNAs that appear in the vast majority of tumours analysed, we found an oncogenic cluster that was over-expressed in 14 tumours, formed by miR-182, miR-96 and miR183, and miR-4746 that was over-expressed in all the tumours analysed (Fig. 3). Conversely, the anti-oncomiRs miR-145, miR-139 and miR-195 were the most strongly down-regulated miRNAs in our dataset. Interestingly, miR145 and miR143, which were also strongly downregulated (see Fig. 3), belong to the tumour-suppressor miRNA cluster miR143/145 implicated in the initial steps of tumorigenesis^15^. Moreover, miR-195 and miR-139 are also well-known tumour suppressor miRNAs^28^.

#### Conserved deregulated miRNA-mRNA target interactions

The 147 genes and 95 miRNAs differentially expressed in the majority of tumour types were used to study potential miRNA-mRNA interactions. As miRNAs commonly repress gene expression through the RNA interference pathway, significant negative correlations in expression were expected to reflect the reliability of possible interactions. Accordingly, only inverse differential expression of miRNA-mRNA pairs predicted from miRGate^29^ were evaluated with a genomic agreement ≥2 (for details see methods).

**Figure 4:**
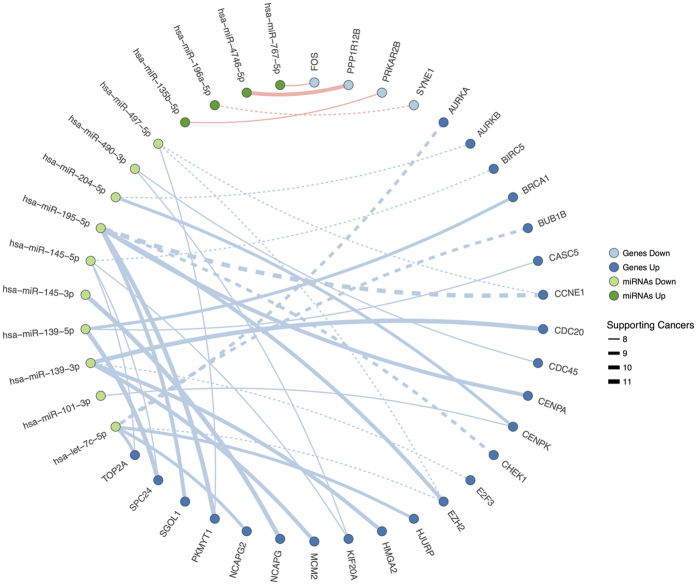
Relevant miRNA-mRNA targets found in the majority of the tumours studied. Lines represent interactions among miRNAs (green) and genes (blue). Continuous line indicates novel interactions, while dashed lines indicate validated interactions.

We analysed the correlation between gene expression and miRNA expression of the predicted pairs using linear regression models that included the log-transformed gene expression values as the response, and the copy number alterations (CNAs), gene methylation (MET) and log-transformed miRNA expression values as explanatory variables (Supplementary Table 11). For each of the 41 significant gene-miRNA pairs, individual models were constructed for every cancer type, and a joint analysis was also performed that included all the samples from all the different types of cancer. In the latter, “cancer type” was also included as an explanatory variable (see methods). Thirty-six out of the 41 pairs (Fig. 4) exhibited a statistically significant negative correlation in the joint analysis (Supplementary Figure 1).

From these 36 pairs, we found 25 novel interactions not described in other specialized databases like OncomirDB^27^, miRCancer^30^ and miRTarBase^31^. Most of these interactions involved cell cycle related genes, like *SPC24* and *CDC20* (regulated by the anti-oncomiR miR-139), and *PKMYT1* (regulated by the tumour suppressor miR-195). The senescence pathway was also represented and while most of the associations identified were known, novel ones were found like *HMGA2*/miR-139 and *EZH2*/miR-195. In the DNA replication pathway, the interaction of the MCM2 gene with the anti-oncomiR miR-139 was notable in the majority of tumours. Finally, the *PRKAR2B*/miR-135b pair was identified that includes the apoptosis related *PRKAR2B* gene.

**Figure 5:**
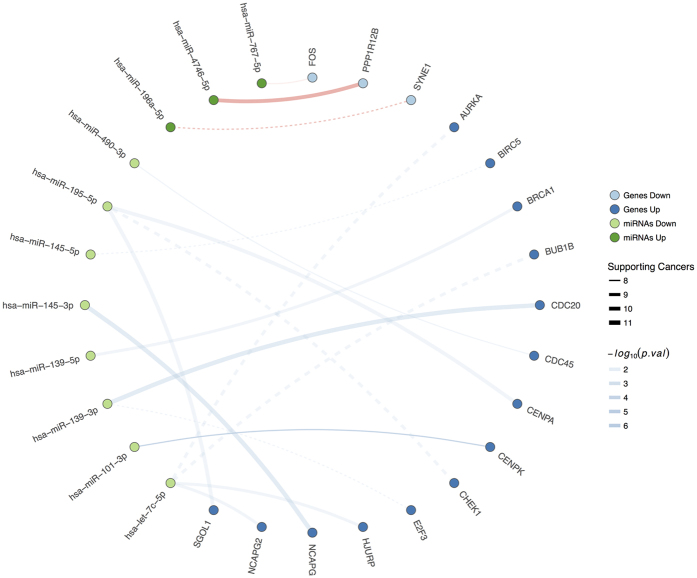
“Highly specific” pairs of interactions. Lines represent interactions among miRNAs (green) and genes (blue). Continuous line indicates novel interactions, while dashed lines indicate validated interactions.

To infer the pairs in which genes are targeted by few miRNAs (henceforth “high specificity” pairs), we identified 17 pairs with least secondary interactions that were positively enriched in the maximum possible number of tumours (see Methods). Interestingly, from these 17 significant pairs (adjusted p-value ≤ 0.05) several downregulated tumour-suppressor miRNAs (e.g., miR-let-7c, miR-139, miR-145 or miR-195) appeared to regulate oncogenic genes related to the cell cycle and DDR pathways (*BUB1B, AURKA, BIRC5, CENPK, BRCA1* and *CHEK1*). Eleven of these 17 resulting pairs had not been proposed previously (Fig. 5 and Supplementary 9). Indeed, these pairs are particularly interesting since the low promiscuity of the miRNA’s interactions favours their potential application as anti-miRNA oligonucleotides (AMOs) targets.

We also observed mRNA-miRNA pairs that only appeared in the two different lung tumour types (LUAD and LUSC). Linear regression models were established for these pairs to account for the CNAs and the MET status of the genes, as indicated above (Supplementary Figure 2, and Supplementary Table 12). We found 40 miRNA-mRNA pairs that involved 35 differentially expressed genes (4 being repressed, see Supplementary Figure 3) and 13 statistically deregulated miRNAs. The genes differentially expressed in these “exclusive lung” pairs were also differentially expressed in other tumours. Moreover, in terms of the 13 miRNAs involved in these lung exclusive interactions, 16 of the 40 associations involve miR-miR-1976, which regulates 16 genes involved in cell cycle, apoptosis and the DDR. The second most strongly represented miRNA in these exclusive lung pairs was miR-let-7b-5p, acting as a regulator in 10 interactions where 8 of the genes implicated also belong to cell cycle pathways, and another 2 to senescence pathways (Supplementary Figure 3). Notably, these two down-regulated miRNAs, which are in the most frequently obtained pairs, are apparently exclusive to these two tumour types as they don’t appear to be significantly repressed in any other tumour.

### Survival Analysis

**Figure 6:**
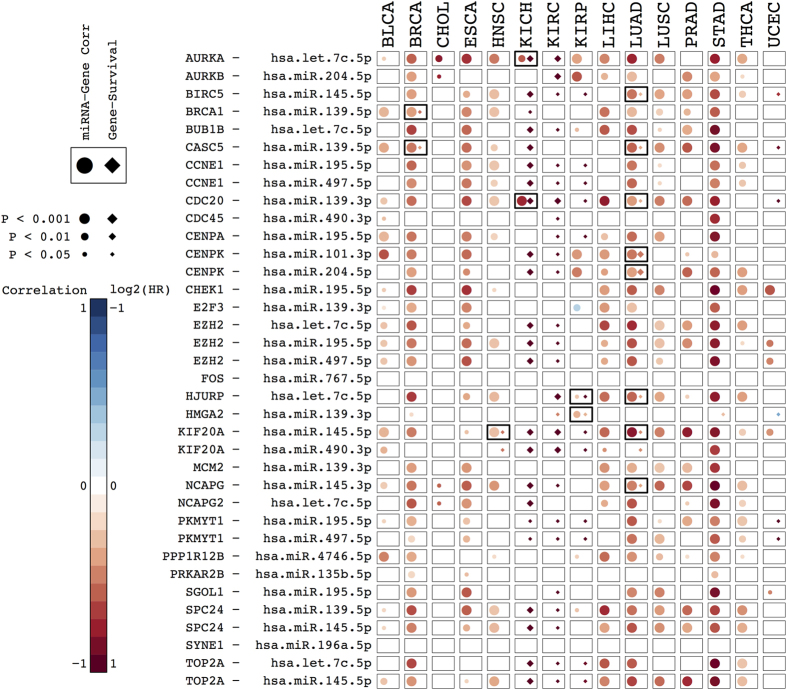
Survival with multifactorial Cox models in 15 tumours. Combined correlation and survival analysis for the selected 36 general miRNA-gene pairs. Every box summarizes the results of the correlation and survival analysis of a miRNA-gene pair (rows) in a particular cancer type (columns). Circles represent miRNA-gene expression correlations, after accounting for CNAs and gene methylation. Negative correlations in red, and positive correlations in blue. Diamonds represent the log2 of the hazard ratio (HR) of Cox proportional hazards models that included tumor stage (or tumor grade, in the case of PRAD) and the log-transformed gene expression values as explanatory variables. Positive log2(HR), i.e. shorter survival, in shades of red. Negative log2(HR), i.e. longer survival, in shades of blue. All P-values were corrected for multi-hypothesis testing by the FDR method. Pairs reaching statistical significance (Adj. P < 0.05) in both miRNA-gen expression correlation and gene expression-survival association are indicated with thicker boxes.

The 36 pairs that exhibited a statistically significant negative correlation in the multivariate linear regression joint analysis (Supplementary Figure 1) were selected for a subsequent survival analyses (Fig. 6). The effect of gene expression on patient survival was explored separately for each cancer type using Cox proportional hazard models that included tumour stage. In these models, both tumour stage (encoded as one of two categories, i.e. Stage I-II vs Stage III-IV) and the log-transformed gene expression values were used as explanatory variables. Information on tumour stage was not available for prostate cancer (PRAD), so in this case tumour grade was employed as the explanatory variable (Gleason score 6–7 vs 8–10). Pairs that exhibited both a significant correlation between miRNA and gene expression, and a significant tumour stage-independent association between gene expression and patient survival, support the hypothesis that miRNA expression regulates gene expression and that the latter affects patient survival. We found miRNA-Gene Survival associations in 11 of the 36 selected pairs.

In 4 pairs, we found significant associations for at least two distinct tumour types. Then, the *HJURP*−miR-let.7c.5p in KIRP and LUAD, the *CDC20*/miR.139.3p in KICH and LUAD, the *KIF20A*/miR145.5.p pair in HNSC and LUAD, and the *CASC5*/miR.139.5p in BRCA and LUAD. The other 7 pairs were found in only one type of cancer each (Fig. 6 and Supplementary Table 13).

We repeated a similar analysis on the lung-exclusive predicted pairs identified in lung adenocarcinomas (LUAD) and squamous cell lung tumours (LUSC). Of the 74 gene-miRNA pairs originally predicted, 40 exhibited a significant negative correlation in the multivariate linear regression joint analysis that included tumour type, CNAs, MET and log-transformed miRNA expression as explanatory variables of gene expression (Supplementary Table 12 and Supplementary Figure 3). Of the 40 lung-exclusive pairs that passed the correlation analysis, we found 14 in which gene expression was also associated with patient survival after accounting for tumour stage (Supplementary Figure 4 and Supplementary Table 14). Notably, 13 were found in LUAD and only one in LUSC.

## Discussion

The identification of miRNA-mRNA interaction pairs is gaining significant attention given their potential implications in devising novel therapies^32^. As such studies pose important experimental challenges, careful computational studies addressing this issue may help to identify novel therapeutic targets, as successfully described elsewhere for individual cancers^33^. A widely used method to evaluate the recurrence of miRNA-target associations is a rank-based method (REC, recurrent score^34^. Like PanMira^35^, this approach uses processed RNASeq and/or microarray data from tumour samples, excluding samples from healthy subjects. By contrast, alternative analyses using TCGA^36^,^37^ also include healthy samples as this provides a more accurate framework to understand target/miRNA relationships in cancer. As such, we have included samples from healthy subjects as controls to identify miRNA/mRNA associations from differentially expressed genes and miRNAs, avoiding other spurious interactions in principle not related to cancer.

Although much work has been done in individual tumours, a systematic analysis to infer conserved miRNA-mRNA interactions in different cancer types based on differential expression has yet to be performed on a large number of raw clinical samples. Thus, to determine if different tumours exhibit distinct miRNA-mRNA interactions, we have studied 15 of the most common tumour types in-depth. We analysed 18,605 RNASeq and miRNASeq raw data samples, following recommended procedures^38^ and applying restrictive filters to reduce false positives.

Our rationale was that miRNAs can act as onco-modulators of relevant target genes influencing their expression in tumours. Thus, by restricting the analyses to relevant cancer pathways, we could identify more suitable candidates. In the pathway analyses we found a general trend towards deregulation, although some genes within the pathways occasionally exhibited the opposite trend, even though the net effect of the remaining genes was to drive an overall “dampening” phenotype.

When considering individual tumour types, our results are consistent with previous findings where individual genes behaved as expected. *SYNE1* is the most significantly repressed gene and its dysregulation has been related to glioblastoma^39^ and ovarian cancer^40^, among others. From the miRNA perspective, we identified important species previously related to tumour progression. For instance, we found miR-4746 to be over-expressed in all the tumours analysed, in accordance with previous studies^41^. Conversely, tumour suppressor miRNAs (miR-145, miR-139 and miR-195) are the most strongly down-regulated miRNAs in our dataset.

To identify suitable relations, as a first filter we only considered de-regulated genes and de-regulated miRNAs that exhibited inverted expression profiles. We next optimized the identification of miRNA binding to precise 3′ UTR sequences that should in principle trigger gene silencing. This effect is well accepted, given that most of the interactions involving miRNAs included the seed regions at 3′ UTRs^42^. To support this observation, additional studies in mammals revealed that there is a tendency to diminish target mRNA levels^11^. However, it is important to note that alternative mechanisms may be at play, including activation due to binding at the promoter or other regions of the gene^43^. Although non-canonical binding sites^42^,^44^ have been described for miRNAs, these are limited and they are not included in most of the available prediction software used here to identify target genes. Gene expression is also influenced by other important “pleiotropic” factors. Moreover, indirect interactions involving complex transcriptional networks of other genes and RNA species may also influence the expression of a given gene. While these influences deserve deeper exploration, their complexity and the lack of reliable data to model these elements in cancer makes this an issue beyond the scope of this work. Thus, for the purposes of simplicity we focus on the most parsimonious explanation, which is gene silencing by binding to 3′ UTR regions.

It is well known that methylation and copy number alterations affect gene expression, especially in cancer. From the original 41 pairs exhibiting significant inverted expression profiles, we filtered out some pairs by accounting for these factors with 36 consistent interactions remaining as both partners appeared to be deregulated together. Of these 36 pairs, 25 had not previously been inferred, although individually most of the genes and miRNAs involved are known to be related to cancer development. An illustrative example is the *SCP24*/miR-139 couplet, where involvement of the individual components is well-established (*SPC24* and miR-139-5p^45^,^46^, while no experimental evidence is available for the pair. A subset of these 36 pairs (17 pairs), appear to be “highly specific” as both partners were not found with many other alternative interactors in the distinct tumour types. These specific associations are noteworthy, as they may be useful for the development of therapies due to the limited off-target effects. From these pairs, 11 interactions had not previously been described, while among associations that had already been experimentally confirmed were those of *AURKA*^47^ and *BUB1B*^48^ with miR-let-7c.

When comparing miRNA-mRNA interactions among tumour types, we observed that tumours of the same origin exhibited interactions not identified in the remaining tumours (Supplementary Figure 3), in particular lung cancers (LUAD and LUSC), and that these were mostly mediated by two types of miRNAs, miR-1976 and let-7b-5p. These miRNAs act as master regulators of genes belonging to the cell cycle and apoptosis pathways. Moreover, they are involved in the majority of these lung exclusive interactions, suggesting a potential value of these miRNAs as biomarkers for lung cancer. In fact miR-1976 is a potential tumour suppressor in lung cancer, as described in relation with the oncogene *PLCE1*^49^.

There are many factors known to affect cancer prognosis. Cancer survival is ultimately the result of a very complex network of mechanisms that include tumour aggressiveness, operability, routine primary treatment, available secondary therapies, possible drug-resistance, patient age, their physical status, comorbidity, etc. All these factors differ radically among cancer types and affect their prognosis profoundly. For many years, clinicians have employed a number of clinical-pathological characteristics that have proven to be very useful to estimate cancer patient survival. Most notably, tumour stage and degree of differentiation have been used as prognostic factors in the diagnosis and management of most cancer types. For instance, patients with Stage III or IV cancers, where the malignant cells have already metastasized to lymph nodes or distant organs, exhibit much worse prognosis than patients with Stage I-II tumours. Similarly, patients with poorly differentiated tumours exhibit worse prognosis than patients with moderately or well-differentiated tumours. Patient age and gender are also typically employed as predictive markers of survival. In recent years, molecular markers of patient survival or of response to treatment have been steadily incorporated into clinical practice (ASCO, https://www.asco.org/practice-guidelines and ESMO, http://www.esmo.org/Guidelines/Guidelines-News). Therefore, a single factor (gene, miRNA) should not be expected to produce a large effect on survival by itself, independently of the many confounding factors that potentially affect patient survival, especially across many different tumour types. As such, caution should be exercised regarding the associations between gene regulation by miRNAs and patient prognosis. Nonetheless, we have recovered known associations in our survival analyses, some of which have been studied extensively in many tumours, like the roles on prognosis of genes *AURKA*^50^,^51^,^52^,^53^ or *BRCA1*^54^,^55^. We also found novel stage-independent prognostic genes not described in the literature like *CASC5*. This gene was previously described in lung cancer^56^, but not in breast cancer, which is the association found in this study. It is worthy to mention that its regulating miRNA (miR-139-5p) has been itself associated to prognosis in lung cancer^57^ but again not in breast. Therefore, our study revealed a novel miRNA-gene-survival association in breast cancer, that is supported by previous findings in lung cancer. Similar findings are the oncogene *BIRC5*, found relevant in LUAD here but only previously described in breast^58^, bladder^59^, chondrosarcoma^60^ and sarcoma^61^. However, its regulating miRNA (miR-145-5p) has been associated to prognosis in gastric^62^ and kidney^63^ tumours. Moreover *CDC20,* described as prognostic in colon^64^ and breast^65^, has been identified as relevant in KICH and LUAD in our study. Finally *NCAPG* and *KIF20A* genes of prognostic value in gliomas^66^,^67^ were found relevant here in LUAD. We also identified a few novel associations in lung tumours (LUAD), for instance *CENPK* has not previously been associated with this cancer type, even though the gene had already been associated to ovarian cancer^68^. When addressing the “exclusive lung interactions” the genes *CDT1, CENPN, DAPK2, HJURP, KIF20A, NACPD2, NCAPG, NCAPH, SMC1B*, and *SPC24* have so far not been associated to survival in lung cancer, although most of its associated miRNAs have been. For instance miR-miR-1976 and miR-let-7b-5p miRNAs had already been associated to prognosis in lung cancer^49^,^69^, and in other tumours like breast^70^, prostate^71^, and gastric^72^. In conclusion, our analysis has revealed novel miRNA-gene correlations that associate with patient prognosis, supported by previous findings on different tumour types.

To summarize, in addition to producing comparable results at the individual level, our integrative analysis provided a confident statistical approach to identify novel miRNA-target relationships in different tumour types within cancer-relevant pathways. The identification of lung exclusive mRNA/miRNA interactions that are significantly associated with survival opens potentially avenues for clinical research into cancers. This exploratory analysis, followed by future experimental validation, could be significant to further decipher the role of miRNAs in tumorigenesis, confirming the validity of our approach to design of miRNA-targeted cancer drugs focusing on the gene/pathway. By confidently identifying specifically altered miRNA-mRNA pairs and interactions, off-target effects could be minimized, reducing side-effects and enhancing already existing treatments.

## Methods

### TCGA Transcriptome samples

A considerable number of tumour types, and the maximum number of control and tumour samples, are necessary to identify miRNA-mRNA associations related to common processes of tumorigenesis. As such, raw data sample files (fastq or bam files) from the TCGA database were used. These restricted samples were obtained by applying to the National Cancer Institute (NCI) and the National Human Genome Research Institute (NHGRI) from the National Institutes of Health (USA) for dbGAP-authorized access. Currently, TCGA includes data on 33 different tumour types obtained from different platforms. Since we are interested in the differential expression of genes and miRNAs implicated in tumour phenotypes, and their interactions, we selected those tumours for which both kinds of transcriptome samples existed. After applying this initial filter, 19 tumour types with over 22,000 paired-end RNASeq and single-end miRNASeq samples were selected. Subsequently, to carry out a comprehensive statistical study by comparing controls with cancer samples, tumours with less than 10 healthy samples and those whose total number of healthy samples did not reach 5% of the total, were discarded. After applying these filters, 15 tumours were selected: Kidney Chromophobe (KICH), Head and Neck squamous cell carcinoma (HNSC), Oesophageal carcinoma (ESCA), Kidney renal papillary cell carcinoma (KIRP), Liver hepatocellular carcinoma (LIHC), Kidney renal clear cell carcinoma (KIRC), Lung Adenocarcinoma (LUAD), Thyroid carcinoma (THAD), Prostate adenocarcinoma (PRAD), Bladder Urothelial Carcinoma (BLCA), Breast invasive carcinoma (BRCA), Lung squamous cell carcinoma (LUSC), Stomach adenocarcinoma (STAD), Cholangiocarcinoma (CHOL) and Uterine Corpus Endometrial carcinoma (UCEC). A total of 18,605 (6,867 RNASeq and 11,738 small-RNASeq) samples were recorded from these tumours and prepared for downloading from December 21^st^ 2015 at the Cancer Genomics Hub (https://cghub.ucsc.edu/). Detailed information about the number of samples for each tumour is shown in Supplementary Table 1.

### Selected pathways

In this study, we selected seven pathways that are highlighted as hallmarks of cancer^4^: the DDR and cell cycle pathways were chosen as characteristic of cancer checkpoints and development; telomere elongation and DNA replication are characteristic of tumour progression; and finally, apoptosis, necrosis and senescence were chosen given their role in inhibiting cancer. A total of 1,264 genes (861 unique) belonging to these pathways were identified, mainly from the Reactome^73^ and KEGG databases^74^. In the case of the DDR pathway, the genes included were obtained from other published studies^75^ and retrieved from the DDRprot database^76^. All gene names and pathways, in which they participate, are shown in Supplementary Table 5.

### Hyper-geometric pathway enrichment

In this work, seven cancer-related pathways were defined and the genes implicated were chosen from three different data sources. Since the classification of our pathways differs from those offered by enrichment analysis methods designed for NGS transcriptome sample analysis, we developed a statistical procedure based on an established approach for pathway analysis of cancer genomes^77^, enabling custom pathways to be integrated. Given our hypergeometric distribution, we used a Fisher’s exact test in a 2 × 2 contingency table to generate an odds ratio (OD) that quantifies the strength of enrichment, as well as a p-value corrected for multiple testing that estimates the proportion of enriched gene sets that would occur by chance given the number of gene sets tested based on the Benjamini-Hochberg method. While an OD >1 for a given pathway means that the number of deregulated genes (either up-regulated or down-regulated) are positively enriched relative to all the genes expressed in the experiment (deregulated genes appear more than expected), an OD <1 means that the given pathway is depleted in altered genes and consequently, it behaves like a healthy sample. As a control, differentially expressed genes equal in size to the number of genes within our described pathway were randomly selected to compare the Ors and adjusted p-values as shown in Supplementary Table 2. Ods for each of the tumour types are shown for any individual pathway in Fig. 2.

### Transcriptome analysis of miRNAs and mRNAs

A total of 11,738 miRNA and 6,787 mRNA experiments were downloaded from the controlled-access Cancer Genomics Hub. These samples were analysed following the protocol proposed previously^38^ and using a pipeline for NGS studies called miARma-Seq^78^. Briefly, this tool brings together well-established software in a single bundle, allowing a complete analysis of the raw data. Quality analysis, adapter removal, read alignment, read count summarizing, differential expression analysis and gene pathway enrichment were performed, among others. For miRNA analysis, we employed miARma-Seq from the miRBase version 20 annotation database^79^, obtaining aligned reads for each mature miRNA. Similarly, the latest GRCh37 EnsEMBL version^80^ was used to acquire the total number of paired aligned reads for each human gene.

### Statistical analysis of the transcriptome samples

To measure the miRNAs or genes differentially expressed in an independent cancer type, we compared the number of aligned reads associated with each miRNA/gene in the control and tumour samples for each individual tumour. To achieve this, edgeR^81^ was used from the differential expression module included in miARma-Seq^78^. As recommended^38^, weakly expressed elements (miRNAs or genes) were filtered out if the CPM (counts per million reads) was <1. Reads were normalized using the TMM method (trimmed mean of M-values) and the exact tests to calculate differences between two groups of negative-binomial counts was performed to gather the log2 fold change, and the Benjamini and Hochberg false discovery rate (FDR) for each miRNA/gene. The miRNAs or genes with a FDR <0.05 were considered significantly and reliably differentially expressed (DE). From these, those with an absolute log2 fold change (log2FC) value >1 were selected for subsequent analyses (Supplementary Tables 6 and 7).

### The miRNA-mRNA target prediction

Differentially expressed genes belonging to our seven defined pathways and miRNAs (FDR <0.05) for each tumour analysed with an absolute log2FC ≥1 were chosen for target prediction. As miRNAs disrupt and reduce the expression of their target mRNAs, we expect miRNAs and mRNAs to exhibit an inverse expression profile. Thus, down-regulated mRNAs for a given tumour were used in the target prediction step for up-regulated miRNAs from the same tumour samples, and vice-versa. For miRNA-mRNA target predictions, we used miRGate^29^, a database of novel predicted miRNA–mRNA pairs computed from a common source of sequences. This repository includes a feature called “genomic agreement” that represents the number of unique predictions obtained from five different methods that occur in the same genomic position. Therefore, they are predicted from different prediction algorithms. To obtain reliable miRNA-mRNA pairs, only targets with an agreement value ≥2 were chosen.

### Integrative analysis of all tumour types

Once all samples for each of the 15 tumour types were analysed, these results were studied in a global framework. As most of the oncogenes and tumour suppressor genes, and their oncomiR and anti-oncomiR regulators, appear to be misexpressed in tumours^82^, statistically deregulated genes and miRNAs (FDR ≤0.05 and log2FC ≥1) were extracted for each tumour type. Moreover, to decrease the number of false positives obtained from prediction algorithms, the miRNA target calculation step was improved by using inverse correlated profiles as recommended elsewhere^20^. Furthermore, genes and miRNAs were selected when both appeared to be differentially expressed in at least eight common tumour types, and only targets with a genomic agreement ≥2 were considered (Supplementary Table 8).

In addition, in order to obtain highly specific miRNA-target associations conserved in the maximum number of tumour types as a representation of a shared deregulated mechanisms in cancer, we applied the following statistical approach: for each partner in a miRNA-mRNA interaction, we computed all the possible deregulated genes and miRNAs, respectively, to obtain the pair that is statistically enriched in the maximum number of tumours with fewer co-interactors. So for each miRNA, we obtained the gene(s) that could be potentially regulated in the maximum number of tumours and conversely, for each gene we obtained regulator miRNA(s) in the maximum number of tumours. This is implemented by computing a 2 × 2 contingency right-tailed Fisher exact test, measuring all existing miRNA-target associations obtained through the differentially expressed genes and miRNAs in the majority of tumours included with a genomic agreement ≥2. P-values from fisher exact test were corrected from multiple testing using the Benjamini-Hochberg method^83^. For details of these pairs see Supplementary Table 9.

### Identification of pairs belonging to the same tumour type

Differentially expressed genes and miRNAs for each individual tumour type were collected (Fig. 1), and like all the aforementioned analysis, we only considered those genes and miRNAs having a FDR < = 0.05 and a significant variation in expression (absolute log2FC >=1). Once we had the set of statistically deregulated genes and miRNAs in each tumour type, we computed all the possible miRNA-mRNA interactions using miRGate^29^ and selecting only those associations predicted by more than one algorithm in the same genomic position in the 3′UTR (genomic agreement >=2). Finally, once we have inferred this set of confident interactions, we first compare them among the 15 tumour types, to obtain a list of exclusive miRNA-mRNA interactions in each tumour type.

### Analyses of methylation and copy number alterations in the pairs identified

The association between gene and miRNA expression was analysed by multifactorial linear regression for every selected gene-miRNA pair, with the gene log-transformed RPKM values as the response and the miRNA log-transformed RPKM as the predictor (Supplementary Tables 11 and 12). To correct for putative confounding factors affecting gene expression, copy number alterations (CNAs) and whole gene methylation (MET) were included in the models. For CNAs, Gistic2-preanalyzed data^84^ was downloaded directly from cBioportal^85^,^86^ and the information was coded as a categorical ordered factor with five different levels: homozygous deletion (−2), hemizygous deletion (−1), no change (0), gain (1) and high level amplification (2). DNA methylation data was obtained from *Wanderer*, a web-based tool that offers convenient and fast access to gene-centred TCGA methylation data^87^. Methylation of cholangiomas (CHOL) was not available from Wanderer at the time. Thus, CHOL methylation data was directly downloaded from the cBioportal. For simplicity, all DNA methylation probes mapping within a particular gene locus were included in its regression models as independent factors. Methylation data was transformed from ß-values to M-values to reduce heterocedasticity before being included in the analyses^88^. All methylation probes used in the analysis are shown in Supplementary Table 10. For each mRNA-miRNA pair, we performed individual regression analyses for every individual cancer type in the study, plus an additional joint analysis that included all cancer types. To account for differences in gene expression and miRNA expression among cancer types, cancer type, and the statistical interaction between cancer type and miRNA expression were included as factors in the joint analyses. In all analyses, the gene-miRNA correlation was calculated as the squared root of the fraction of variance in gene expression explained by miRNA expression obtained by ANOVA with Type-III sum of squares, after accounting for the other factors, i.e.: cancer type and its interaction with miRNA expression (where applicable), CNAs and MET. The direction of the correlation, either positive or negative, as well as the *t*-statistic and the resulting P-value were calculated from the coefficients of the linear models. P-values were adjusted by the FDR method, taking into consideration that every mRNA-miRNA association was analysed in 16 different datasets^83^. Regression could not be calculated in some cases where either the gene or the miRNA were not detected in the RNASeq data of a particular tumour type. In those cases, the correlation between gene and miRNA in that tumour type was considered to be non-existent. Details are available in Supplementary Tables 8 and 1, Supplementary Figures 1 and 2.Only pairs exhibiting negative correlation between gene and miRNA expression, and with a P value < 0.05 after multi-hypothesis correction in the joint analysis, were selected for subsequent analyses (Supplementary Tables 11 and 12).

### Survival analysis

For each gene-miRNA pair, the effect of gene and miRNA expression on patient survival was analysed by Cox’s proportional hazards modelling separately in every cancer type. All the models included tumour stage as a co-factor, coded into two categories: good prognosis (stages I or II) and bad prognosis (stages III, IV and X). In prostate cancer, the classification was performed based on tumour grade instead of tumour stage, where good prognosis involved tumours with Gleason score of 6 or 7, and bad prognosis tumours with Gleason score ≥ 8. In these analyses, gene or miRNA expression values were introduced as quantitative log-transformed variables. Multivariate Cox proportional hazards analysis provides an estimate of the effect of the gene/miRNA expression on survival, independently of the effect of tumour stage (or tumour grade, in the case of prostate cancer). P-values were adjusted by the FDR method^83^ and survival analyses are shown in Fig. 6 and Supplementary Figure 4. For details see Supplementary Tables 13 and 14.

The code to analyse the data can be found at https://github.com/amrojasmendoza/mRNA-miRNAs-cancer-pathways.

## Additional Information

**How to cite this article**: Andrés-León, E. *et al*. Novel miRNA-mRNA interactions conserved in essential cancer pathways. *Sci. Rep.* **7**, 46101; doi: 10.1038/srep46101 (2017).

**Publisher's note:** Springer Nature remains neutral with regard to jurisdictional claims in published maps and institutional affiliations.

## Supplementary Material

Supplementary Information

Supplementary Table 2

Supplementary Table 3

Supplementary Table 4

Supplementary Table 5

Supplementary Table 6

Supplementary Table 7

Supplementary Table 8

Supplementary Table 9

Supplementary Table 10

Supplementary Table 11

Supplementary Table 12

Supplementary Table 13

Supplementary Table 14
