# Supplementary material for: Novel miRNA-mRNA interactions conserved in essential cancer pathways
Source: Sci Rep. 2017 Apr 7;7:46101. doi: 10.1038/srep46101 (PMC5384238; doi:10.1038/srep46101)
Supplement: Supplementary Information [file srep46101-s1.pdf]

Novel miRNA-mRNA interactions conserved in essential cancer pathways

Eduardo Andrés-León[1#], Ildefonso Cases[2] , Sergio Alonso[*3], and Ana M. Rojas[1*]

[1]Computational Biology and Bioinformatics Group, Institute of Biomedicine of Seville, HUVR/US/CSIC, 41013 Sevilla, Spain.

[2]REDgene Bioinformatics, Sevilla, Spain.

[3]Program of Predictive and Personalized Medicine of Cancer (PMPPC), Germans Trias i Pujol Research Institute (IGTP), Can Ruti Campus, Badalona, Barcelona, Spain

[*]corresponding authors.

SUPPLEMENTARY INFORMATION

SUPPLEMENTARY TEXT 2

Supplementary Figures 7

Supplementary Figure 1. Effect of CNAs and methylation in expression 7

Supplementary Figure 2. Effect of CNAs and MET in pairs identified in lung 8

Supplementary Figure 3. Network interactions in two lung tumour types. 9

Supplementary Figure 4. Survival with multifactorial Cox models in lung. 10

Supplementary tables. 11

Supplementary Table 1. TGCA samples included in this work. 11

Supplementary Table 2 (legend). Statistically significant deregulation of pathways for each tumor 11

Supplementary Table 3 (legend). Threshold selection for tumor number (genes) 11

Supplementary Table 4 (legend). Threshold selection for tumor number (miRNAs). 11

Supplementary Table 5 (legend). Selected genes from cancer-related pathways. 12

Supplementary Table 6 (legend). Differentially expressed pathways- genes 12

Supplementary Table 7 (legend). Differentially expressed miRNAs 12

Supplementary Table 8 (legend). Consistent miRNAs-mRNAs across tumors. 12

Supplementary Table 9 (legend). High specific miRNAs-mRNAs across tumours 12

Supplementary Table 10 (legend). Methylation probes for linear models 13

Supplementary Table 11 (legend). Gene-miRNA correlation analysis of 41 general pairs.. 13

Supplementary Table 12 (legend). gene-miRNA correlaytion analysis of 74 lung-exclusive pairs 13

Supplementary Table 13 (legend). Survival data for the general pairs 13

Supplementary Table 14 (legend). Survival data for lung pairs 13

SUPPLEMENTARY TEXT

Transcriptome analysis of individual tumours.

RNAseq data from more than eleven thousand normal and tumour samples obtained from cancer patients with 15 different tumour types were included in the study (see Supplementary Table 1 for details). Differential expression analyses were performed to identify deregulated genes and miRNAs. Only those exhibiting reliable FDR values (FDR<0.05) and significantly altered expression (absolute log2FC greater than 1) were selected for further analysis. Kidney renal clear cell (KIRC) and bladder carcinoma (BLCA) were the two tumours with more differentially expressed genes and miRNAs (334 and 399, respectively). In contrast, Thyroid (THCA) and Prostate carcinomas (PRAD) had the lowest number of altered genes and miRNAs (61 genes in both types of tumour, and 94 and 97 miRNAs, respectively).

Pathway enrichment: We next studied the enrichment of these differentially expressed genes to identify relevant pathways (Fisher adjusted p-value <0.05: see methods). ESCA, BLCA, BRCA, LIHC and LUSC were the tumour types with more positively enriched pathways (Figure 2). Odd ratios and p-values for each pathway and tumour type are shown in Supplementary table 2.

When studying differentially expressed genes according to the pathway in which they act, ESCA, BLCA, BRCA, LIHC and LUSC were the tumour types with the highest number of differentially expressed genes in pathways such as DNA replication, cell cycle and telomere elongation, thereby promoting proliferation compared to normal cells. Interestingly, the GLOBOCAN study carried out by the World Health Organization, designated lung, liver, stomach, breast and oesophagus tumours as among the most aggressive cancers due to their high rate of mortality (1). When comparing the pathways in all cancer types, we inferred that pathways related to cancer inhibition

(apoptosis, necrosis and senescence or DDR) were frequently depleted in differentially expressed genes, a striking trend for apoptosis in 14 of the 15 tumours studied. This is consistent with the fact that avoiding programmed cell death through the inhibition of cell death signals is one of the hallmarks in cancer biology (2).

Gene differential expression analysis: The deregulated genes identified in an analysis of 11,738 samples from 15 individual tumour types were studied in detail (see methods). The number of deregulated genes is shown in Figure 1 where 228 were identified in BLCA samples, 212 genes in BRCA and 175 in STAD. In these types of cancer, the deregulation of the tumour suppressor *GSN* (Gelsolin) exhibited the highest statistical significance. In CHOL samples, 260 deregulated genes were identified. Among them. deregulation of *CD14* exhibited the highest statistical significance. In ESCA samples, 208 genes were altered and *MYPT2/PPP1R12B* was the most significantly deregulated.

In KICH, KIRC and KIRP kidney tumour samples, the expression of 126, 143 and 334 genes were found differentially expressed, respectively. *HSPA2* was commonly altered in kidney tumours and *TAU* (*MAPT*) was one of the most strongly deregulated genes of the 154 genes altered in HNSC. In hepatocellular carcinomas (LIHC), the deregulation of 192 genes was significant, being *UBE2T* the one exhibiting the highest statistical significance. In the case of lung tumours (LUAD and LUSC), *TUBB1* was the most strongly deregulated gene of the 198 and 202 genes in these tumour samples, respectively. By contrast, *UNC5B* was the most statistically significant deregulated gene in prostate adenocarcinoma (PRAD) of the 79 deregulated genes identified. Of the 46 statistically altered genes identified in Thyroid tumours, *BBC3* (also known as *PUMA*) showed the highest statistically significant deregulation. Finally, from the 292 genes in UCEC, *SYNE* was the most statistically significant deregulated gene. All details about

the differentially expressed genes in each tumour type are shown in Supplementary Table 6.

When considering individual tumour types, our results are consistent with previous findings. Most of our inferred over-expressed genes are known oncogenes, and the majority of the inhibited genes are tumour suppressors. Indeed, the tumour suppressor *GSN* (Gelsolin) was the found repressed in bladder (BLAC), breast (BRCA) and stomach (STAD) tumours, as seen elsewhere (3-5). *CD14* in cholangiocarcinoma, *MYPT2* in oesophageal carcinoma and *UNC5B* in prostate tumours are all known markers for cell invasion (6-8). In kidney cancers, *HSPA2* was the most commonly altered gene in KICH, KIRC and KIRP tumours, where its repression has been related to cancer growth and metastasis (9). *TAU* (*MAPT*), the most deregulated gene in head and neck (HNSC) tumours, is known to be related to apoptosis and *TP53* expression (10). In hepatocellular carcinomas (LICH), however, the most significant gene was *UBE2T*, whose over-expression has been found to be associated with oncogenic properties such as tumour growth and metastatic invasion in liver patients (11). In the case of lung tumours (LUAD and LUSC), the most significantly deregulated gene in both types of cancer was *TUBB1* (Tubulin Beta 1), which encodes for a GTPase protein involved in microtubules formation. The role of microtubules in cancer is known (12) and some authors have confirmed a relationship among tubulin and resistance to chemotherapy in lung cancer (13). In thyroid tumours, *BBC3* (also known as *PUMA*) was the most severely deregulated gene. This gene has been found to be implicated in cell proliferation (14). Finally, *SYNE1* was the most strongly deregulated gene in uterine cancer, consistent with previous findings (15).

miRNA differential expression analysis: Differential expression analysis of 6,867 small RNASeq samples from 15 different tumours types was also performed

(summarized in Figure 1). Briefly, as previously found when comparing gene transcriptional data, BLCA, BRCA and STAD exhibited similar miRNA expression profiles. Thus, several tumour suppressor miRNAs were apparently repressed, such as miR-139, miR-143/145 or miR-195. Other tumour suppressor miRNAs that were inhibited, as well as over-expressed oncomiRs, were found in the remaining tumour types. Among these, the oncomiRs miR-182 in CHOL, miR-148a in ESCA, miR-381 in HNSC were particularly noteworthy, as were the anti-oncomiR miR-184, miR-222 and miR-508 in KIRCH, KIRP and KIRC, respectively. MiR-424 was the most significantly deregulated miRNA in hepatocellular carcinoma. In lung cancer, repression of the tumour suppressor miRNAs, miR-139 (in LUAD) and miR-30d (in LUSC), was evident. In PRAD, miR-891a from the oncogenic miR-888 cluster was the most statistically significant over-expressed miRNA. In contrast, the most statistically significant deregulated miRNAs in THCA were miR-4709 and miR-146b. Tumour suppressor miR-202 was found downregulated in uterine tumours. For details regarding the differentially expressed miRNAs in each tumour type, please refer to Supplementary Table 7.

Our results revealed that most of the up-regulated miRNAs can be classified as oncomiRs (miR-184, miR-891a from the miR-888 cluster, or miR-96 from the miR-183 cluster), while tumour-suppressor miRNAs tend to be inhibited. Indeed, miR-139, miR-143/145 and miR-195 were the most strongly repressed miRNAs in BRCA, BLCA and STAD, as previously described (16-18), and the miR-381 species were the most differentially expressed miRNA in HNSC, consistent with previous findings (19). Silencing of miR-424 in hepatocellular carcinoma has been associated with accelerated cell proliferation, migration and invasion (23). In lung cancer, we found the tumour suppressor miRNAs miR-139 and miR-30d repressed in LUAD and LUSC, respectively, which is consistent with previous findings (24, 25). The miR-891a of the oncogenic

miR-888 cluster was the most strongly altered miRNA in prostate cancer. This miRNA has been related to prostate cancer growth (26). In thyroid tumours, the most strongly deregulated miRNAs were miR-4709 and miR-146b, as previously reported (27). The tumour suppressor miR-202 was the most strongly inhibited in uterine tumours. This miRNA has already been linked with endometrial carcinoma (28).

Supplementary Figures

Supplementary Figure 1. Effect of CNAs and methylation in expression .

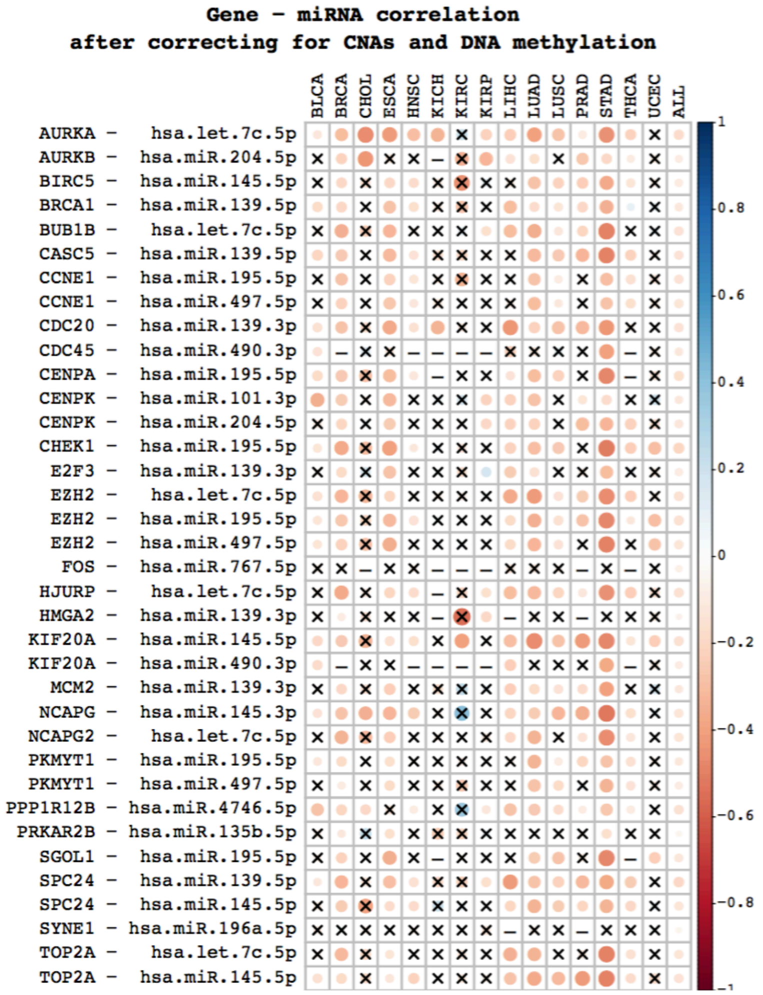

Correlation between gene expression and miRNA expression in the general gene-miRNA pairs. Correlation was analyzed by multifactorial lineal regression containing CNAs, gene methylation and miRNA expression as explanatory variables of gene expression. These analyses were performed individually for every gene-miRNA pair (rows) in every cancer type (columns), and in a joint analysis on all cancer types (column ALL) that also contained cancer type as explanatory variable. The size of the circles is proportional to absolute value of the correlation. Positive correlations are in blue. Negative correlations are in red. Regression models that did not reach statistical significance after FDR-multihypothesis correction ($P<0.05$) are marked with crosses. Models where either gen expression or miRNA expression was not detected are indicated with a dash.

Supplementary Figure 2. Effect of CNAs and MET in pairs identified in lung

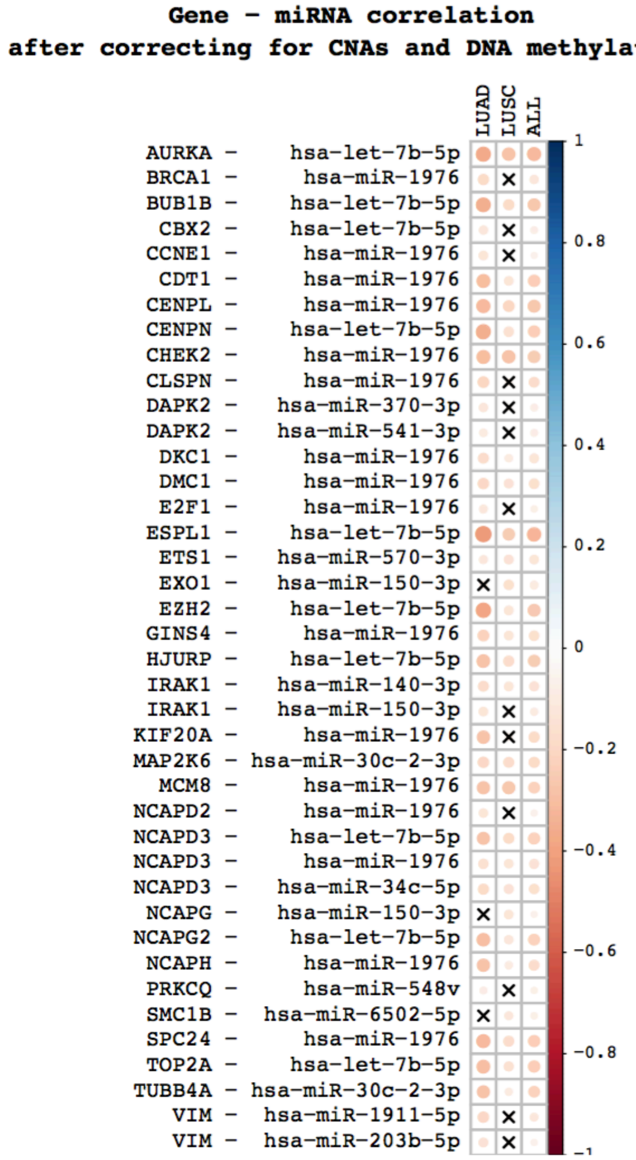

Correlation between gene expression and miRNA expression in the lung exclusive gene-miRNA pairs. Correlation was analyzed by multifactorial lineal regression containing CNAs, gene methylation and miRNA expression as explanatory variables of gene expression. These analyses were performed individually for every gene-miRNA pair (rows) in lung adenocarcinomas (LUAD) and lung squamous cancers (LUSC), and in a joint analysis on both cancer types (ALL) that also contained cancer type as explanatory variable. The size of the circles is proportional to absolute value of the correlation. Positive correlations are in blue. Negative correlations are in red. Regression models that did not reach statistical significance after FDR-multihypothesis correction ($P<0.05$) are marked with crosses. Models where either gen expression or miRNA expression was not detected are indicated with a dash..

Supplementary Figure 3. Network interactions in two lung tumour types.

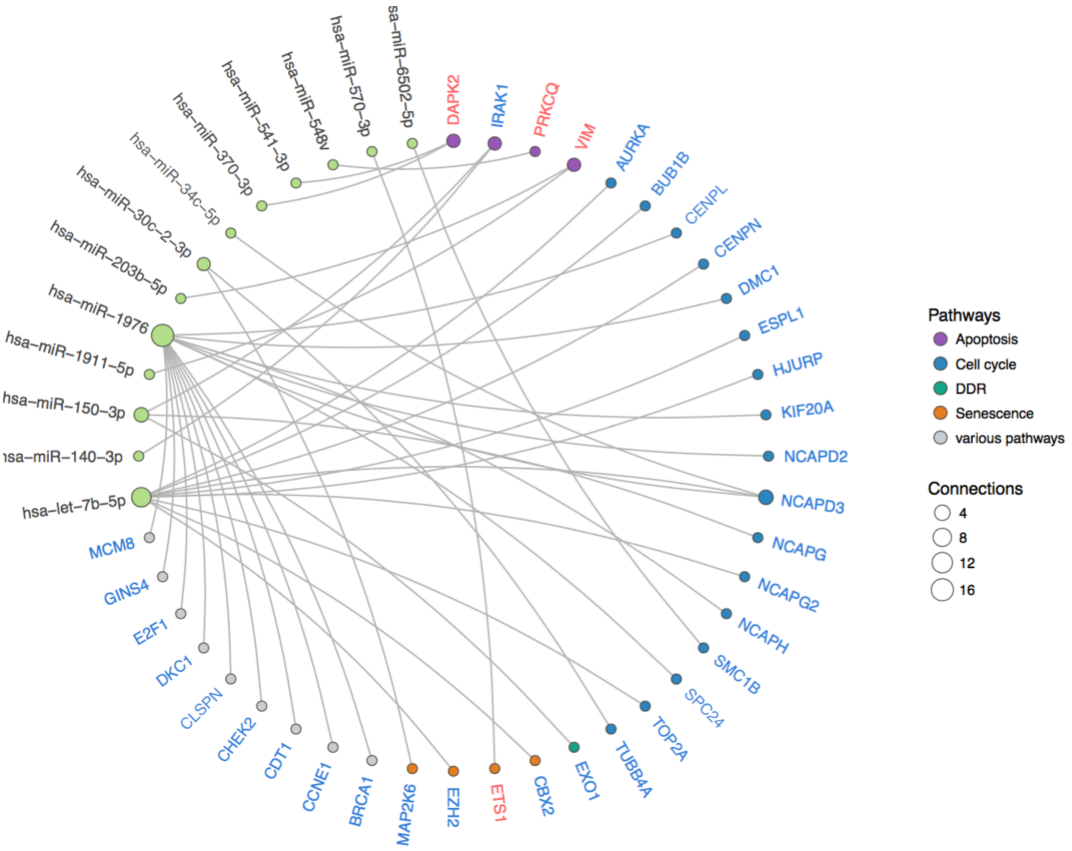

Lung cancer (LUAD and LUSC) gene-miRNA interaction network. Blue fonts indicate activation, while red repression. The diameter of the circles indicates the number of established connections, and their colour indicate the pathway in which these genes participate.

Supplementary Figure 4. Survival with multifactorial Cox models in lung.

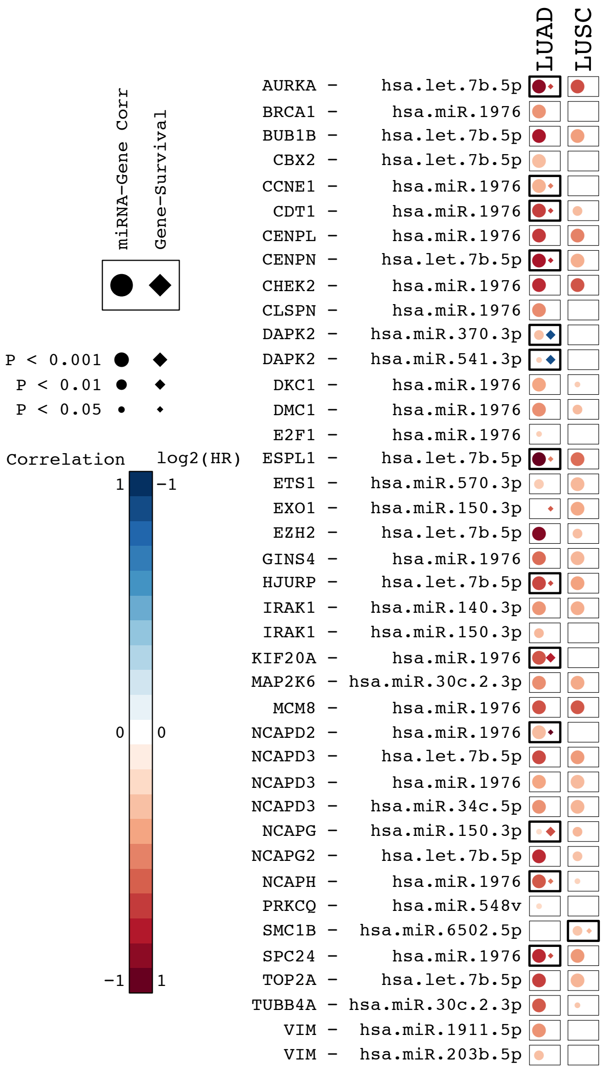

Combined correlation and survival analysis for the selected 40 lung-exclusive miRNA-gene pairs. Every box summarizes the results of the correlation and survival analysis of a miRNA-gene pair (rows) in a particular cancer type (columns). Circles represent miRNA-gene expression correlations, after accounting for CNAs and gene methylation. In red, negative correlations. In blue, positive correlations. Diamonds represent the log2 of the hazard ratio (HR) of Cox proportional hazards models that included tumor stage and the log-transformed gene expression values as explanatory variables. Positive log2(HR), i.e. shorter survival, in shades of red. Negative log2(HR), i.e. longer survival, in shades of blue. All P-values were corrected for multi-hypothesis testing by the FDR method. Pairs reaching statistical significance (Adj. P<0.05) in both miRNA-gen expression correlation and gene expression-survival association, are indicated with thicker boxes.

Supplementary tables.

Supplementary Table 1. TGCA samples included in this work.

Control and tumour samples are specified for each of the 15 tumours analysed.

		RNASeq			miRNASeq		
**Tumour**	**Acronym**	**Control**	**Tumour**	**% control**	**Control**	**Tumour**	**% control**
Kidney Chromophobe	KICH	25	66	37.88%	25	66	37.88%
Head and Neck squamous cell carcinoma	HNSC	39	472	8.26%	83	849	9.78%
Esophageal carcinoma	ESCA	13	184	7.07%	13	186	6.99%
Kidney renal papillary cell carcinoma	KIRP	32	240	13.33%	60	375	16.00%
Liver hepatocellular carcinoma	LIHC	50	268	18.66%	99	460	21.52%
Kidney renal clear cell carcinoma	KIRC	72	981	7.34%	142	1048	13.55%
Lung Adenocarcinoma	LUAD	58	513	11.31%	92	923	9.97%
Thyroid carcinoma	THAD	59	498	11.85%	118	910	12.97%
Prostate adenocarcinoma	PRAD	41	333	12.31%	102	635	16.06%
Bladder Urothelial Carcinoma	BLCA	19	414	4.59%	36	495	7.27%
Breast invasive carcinoma	BRCA	113	1134	9.96%	207	2000	10.35%
Lung squamous cell carcinoma	LUSC	50	490	10.20%	90	825	10.91%
Stomach adenocarcinoma	STAD	35	415	8.43%	74	683	10.83%
Cholangiocarcinoma	CHOL	9	36	25.00%	9	36	25.00%
Uterine Corpus Endometrial carcinoma	UCEC	39	169	23.08%	62	1035	5.99%
		**Control**	**Tumour**	**RNASeq**	**Control**	**Tumour**	**miRNASeq**
**Totals**		654	6213	**6867**	1212	10526	**11738**

Supplementary Table 2 (legend). Statistically significant deregulation of pathways for each tumor

This table is available as a separate file. The table includes pathway name, probability value and odd ratio of genes belonging to the pathway, and probability value and odd ratio of random genes used as control. Those in blue are statistically significant (Pvalue<0.05).

Supplementary Table 3 (legend). Threshold selection for tumor number (genes).

This table is available as a separate file. Cut-off selection for cancer-related genes. Different thresholds were calculated in order to obtain the most enriched list of genes. The table displays the cutoff used, the total number of genes found at this threshold, the number of cancer related genes found in the obtained list, the false discovery rate, the odd ratio and the database used to obtain the list of known cancer related gene.

Supplementary Table 4 (legend). Threshold selection for tumor number (miRNAs).

This table is available as a separate file. Cut-off selection for cancer-related miRNAs. Different threshold were calculated in order to obtain the most enriched list of cancer miRNA. The table

displays the cutoff used, the total number of miRNAs found at this threshold, the number of cancer related miRNAs found in the obtained list, the false discovery rate, the odd ratio and the database used to obtain the list of known cancer related miRNAs.

Supplementary Table 5 (legend). Selected genes from cancer-related pathways.

This table is available as a separate file. Selected genes from cancer-related pathways. Genes included in this analysis in each pathway and their corresponding source

Supplementary Table 6 (legend). Differentially expressed pathways- genes.

This table is available as a separate file. Differentially expressed pathways-genes. Differentially expressed genes from relevant pathways in each tumour and in the selected threshold. Every tumour-type table displays gene name, Pathway name, log2FC (tumor/control sample), log2 counts per million (logCPM), probability value and the false discovery rate (FDR) adjusted probability value.

Supplementary Table 7 (legend). Differentially expressed miRNAs

This table is available as a separate file. Differentially expressed miRNAs. Differentially expressed miRNAs from each tumour type at the selected threshold. All tumor type tables display miRNA identifier, log2FC (tumor/control sample), log2 counts per million, probability value and the false discovery rate (FDR) adjusted probability value.

Supplementary Table 8 (legend). Consistent miRNAs-mRNAs across tumours.

This table is available as a separate file. Consistent miRNAs-mRNAs across tumors. Gene-miRNA relationships conserved in the majority of the tumours. The table includes gene pathway, gene name, miRNA name, genomic agreement, total number of tumours where the pair was conserved, gene and miRNA expression (+ indicates over-expressed and – repressed) tumor names and a reference (if available) of the validated pair. Kidney Chromophobe (KICH), Head and Neck squamous cell carcinoma (HNSC), Esophageal carcinoma (ESCA), Kidney renal papillary cell carcinoma (KIRP), Liver hepatocellular carcinoma (LIHC), Kidney renal clear cell carcinoma (KIRC), Lung Adenocarcinoma (LUAD), Thyroid carcinoma (THAD), Prostate adenocarcinoma (PRAD), Bladder Urothelial Carcinoma (BLCA), Breast invasive carcinoma (BRCA), Lung squamous cell carcinoma (LUSC), Stomach adenocarcinoma (STAD), Cholangiocarcinoma (CHOL) and Uterine Corpus Endometrial carcinoma (UCEC).

Supplementary Table 9 (legend). High specific miRNAs-mRNAs across tumours

This table is available as a separate file. Specific gene-miRNA pairs in the majority of the tumours. It includes gene names, gene expression (+ indicates over-expressed and – indicates repressed), pathway, miRNA name, miRNA expression (+ indicates over-expressed and – indicates repressed), total number of tumours where this pair is conserved, probability value and experimentally verified reference if found. The name of the tumors are: Kidney Chromophobe

(KICH), Head and Neck squamous cell carcinoma (HNSC), Esophageal carcinoma (ESCA), Kidney renal papillary cell carcinoma (KIRP), Liver hepatocellular carcinoma (LIHC), Kidney renal clear cell carcinoma (KIRC), Lung Adenocarcinoma (LUAD), Thyroid carcinoma (THAD), Prostate adenocarcinoma (PRAD), Bladder Urothelial Carcinoma (BLCA), Breast invasive carcinoma (BRCA), Lung squamous cell carcinoma (LUSC), Stomach adenocarcinoma (STAD), Cholangiocarcinoma (CHOL) and Uterine Corpus Endometrial carcinoma (UCEC).

Supplementary Table 10 (legend). Methylation probes for linear models

This table is available as a separate file. Methylation probes for linear models. Illumina HM450K methylation probes employed in this study. For every probe, the genetic position of the interrogated CpG site and the associated gene are indicated.

Supplementary Table 11 (legend). Gene-miRNA correlation analysis of 41 general pairs

This table is available as a separate file. Correlation coefficients and p-vales of multifactorial lineal model regression analyses using tumour type (when applicable), CNAs, methylation and log-transformed miRNA expression as explanatory variables, and log-transformed gene espression as response, for every general gene-miRNA pair (rows) analyzed individually per tumour type (columns), and in a joint analysis comprising all tumour samples (column ALL).

Supplementary Table 12 (legend). gene-miRNA correlaytion analysis of 74 lung-exclusive pairs

This table is available as a separate file. Correlation coefficients and p-vales of multifactorial lineal model regression analyses using tumour type (when applicable), CNAs, methylation and log-transformed miRNA expression as explanatory variables, and log-transformed gene espression as response, for every lung-exclusive gene-miRNA pair (rows) analyzed individually per tumour type (columns), and in a joint analysis comprising all tumour samples (column ALL).

Supplementary Table 13 (legend). Survival data for the general pairs

This table is available as a separate file. Survival data for the general pairs. Cox proportional hazard coefficients and p-values of gene expression vs survival in general gene-miRNA pairs. The coefficients were calculated by multifactorial Cox proportional hazard that included tumour stage (or grade, for PRAD) and log-transformed gene expression values as explanatory variables for survival.

Supplementary Table 14 (legend). Survival data for lung pairs

This table is available as a separate file. Survival data for lung pairs. Cox proportional hazard coefficients and p-values of gene expression vs survival in lung-exclusive gene-miRNA pairs. The coefficients were calculated by multifactorial Cox proportional hazard that included tumour stage (or grade, for PRAD) and log-transformed gene expression values as explanatory variables for survival.

.

REFERENCES

1. Ferlay J, Soerjomataram I, Dikshit R, Eser S, Mathers C, Rebelo M, et al. Cancer incidence and mortality worldwide: sources, methods and major patterns in GLOBOCAN 2012. Int J Cancer. 2015;136(5):E359-86.
2. Hanahan D, Weinberg RA. Hallmarks of cancer: the next generation. Cell. 2011;144(5):646-74.
3. Tanaka M, Mullauer L, Ogiso Y, Fujita H, Moriya S, Furuuchi K, et al. Gelsolin: a candidate for suppressor of human bladder cancer. Cancer Res. 1995;55(15):3228-32.
4. Winston JS, Asch HL, Zhang PJ, Edge SB, Hyland A, Asch BB. Downregulation of gelsolin correlates with the progression to breast carcinoma. Breast Cancer Res Treat. 2001;65(1):11-21.
5. Kuzumaki N, Tanaka M, Sakai N, Fujita H. [Tumor suppressive function of gelsolin]. Gan To Kagaku Ryoho. 1997;24(11):1436-41.
6. Subimerb C, Pinlaor S, Lulitanond V, Khuntikeo N, Okada S, McGrath MS, et al. Circulating CD14(+) CD16(+) monocyte levels predict tissue invasive character of cholangiocarcinoma. Clin Exp Immunol. 2010;161(3):471-9.
7. Li YR, Yang WX. Myosins as fundamental components during tumorigenesis: diverse and indispensable. Oncotarget. 2016.
8. Kong CZ, Liu J, Liu L, Zhang Z, Guo KF. Interactional expression of netrin-1 and its dependence receptor UNC5B in prostate carcinoma. Tumour Biol. 2013;34(5):2765-72.
9. Singh S, Suri A. Targeting the testis-specific heat-shock protein 70-2 (HSP70-2) reduces cellular growth, migration, and invasion in renal cell carcinoma cells. Tumour Biol. 2014;35(12):12695-706.
10. Wang HH, Li HL, Liu R, Zhang Y, Liao K, Wang Q, et al. Tau overexpression inhibits cell apoptosis with the mechanisms involving multiple viability-related factors. J Alzheimers Dis. 2010;21(1):167-79.
11. Wen M, Kwon Y, Wang Y, Mao JH, Wei G. Elevated expression of UBE2T exhibits oncogenic properties in human prostate cancer. Oncotarget. 2015;6(28):25226-39.
12. Parker AL, Kavallaris M, McCarroll JA. Microtubules and their role in cellular stress in cancer. Front Oncol. 2014;4:153.
13. Dumontet C, Isaac S, Souquet PJ, Bejui-Thivolet F, Pacheco Y, Peloux N, et al. Expression of class III beta tubulin in non-small cell lung cancer is correlated with resistance to taxane chemotherapy. Bull Cancer. 2005;92(2):E25-30.
14. Yin Q, Liu S, Dong A, Mi X, Hao F, Zhang K. Targeting Transforming Growth Factor-Beta1 (TGF-beta1) Inhibits Tumorigenesis of Anaplastic Thyroid Carcinoma Cells Through ERK1/2-NFkappakB-PUMA Signaling. Med Sci Monit. 2016;22:2267-77.
15. O'Mara TA, Glubb DM, Painter JN, Cheng T, Dennis J, Australian National Endometrial Cancer Study G, et al. Comprehensive genetic assessment of the ESR1

locus identifies a risk region for endometrial cancer. Endocr Relat Cancer. 2015;22(5):851-61.
16. Yonemori M, Seki N, Yoshino H, Matsushita R, Miyamoto K, Nakagawa M, et al. Dual tumor-suppressors miR-139-5p and miR-139-3p targeting matrix metalloprotease 11 (MMP11) in bladder cancer. Cancer Sci. 2016.
17. Das AV, Pillai RM. Implications of miR cluster 143/145 as universal anti-oncomiRs and their dysregulation during tumorigenesis. Cancer Cell Int. 2015;15:92.
18. Wu WY, Xue XY, Chen ZJ, Han SL, Huang YP, Zhang LF, et al. Potentially predictive microRNAs of gastric cancer with metastasis to lymph node. World J Gastroenterol. 2011;17(31):3645-51.
19. Lajer CB, Garnaes E, Friis-Hansen L, Norrild B, Therkildsen MH, Glud M, et al. The role of miRNAs in human papilloma virus (HPV)-associated cancers: bridging between HPV-related head and neck cancer and cervical cancer. Br J Cancer. 2012;106(9):1526-34.
20. Su Z, Chen D, Li Y, Zhang E, Yu Z, Chen T, et al. microRNA-184 functions as tumor suppressor in renal cell carcinoma. Exp Ther Med. 2015;9(3):961-6.
21. Khella HW, Butz H, Ding Q, Rotondo F, Evans KR, Kupchak P, et al. miR-221/222 Are Involved in Response to Sunitinib Treatment in Metastatic Renal Cell Carcinoma. Mol Ther. 2015;23(11):1748-58.
22. Zhai Q, Zhou L, Zhao C, Wan J, Yu Z, Guo X, et al. Identification of miR-508-3p and miR-509-3p that are associated with cell invasion and migration and involved in the apoptosis of renal cell carcinoma. Biochem Biophys Res Commun. 2012;419(4):621-6.
23. Yu L, Ding GF, He C, Sun L, Jiang Y, Zhu L. MicroRNA-424 is down-regulated in hepatocellular carcinoma and suppresses cell migration and invasion through c-Myb. PLoS One. 2014;9(3):e91661.
24. Xu W, Hang M, Yuan CY, Wu FL, Chen SB, Xue K. MicroRNA-139-5p inhibits cell proliferation and invasion by targeting insulin-like growth factor 1 receptor in human non-small cell lung cancer. Int J Clin Exp Pathol. 2015;8(4):3864-70.
25. Chen D, Guo W, Qiu Z, Wang Q, Li Y, Liang L, et al. MicroRNA-30d-5p inhibits tumour cell proliferation and motility by directly targeting CCNE2 in non-small cell lung cancer. Cancer Lett. 2015;362(2):208-17.
26. Lewis H, Lance R, Troyer D, Beydoun H, Hadley M, Orians J, et al. miR-888 is an expressed prostatic secretions-derived microRNA that promotes prostate cell growth and migration. Cell Cycle. 2014;13(2):227-39.
27. Li X, Abdel-Mageed AB, Mondal D, Kandil E. MicroRNA expression profiles in differentiated thyroid cancer, a review. Int J Clin Exp Med. 2013;6(1):74-80.
28. Hiroki E, Akahira J, Suzuki F, Nagase S, Ito K, Suzuki T, et al. Changes in microRNA expression levels correlate with clinicopathological features and prognoses in endometrial serous adenocarcinomas. Cancer Sci. 2010;101(1):241-9.
